# A Bioluminescent Sensor for Rapid Detection of PPEP-1, a *Clostridioides difficile* Biomarker

**DOI:** 10.3390/s21227485

**Published:** 2021-11-11

**Authors:** Kevin K. Ng, Zachary E. Reinert, Jeroen Corver, Danica Resurreccion, Paul J. Hensbergen, Jennifer A. Prescher

**Affiliations:** 1 Department of Pharmaceutical Sciences, University of California, Irvine, CA 92697, USA; kkng@uci.edu; 2 Department of Chemistry, University of California, Irvine, CA 92697, USA; zreinert@gmail.com; 3 Section Experimental Bacteriology, Department of Medical Microbiology, Leiden University Medical Center, Leiden University, 2333 ZA Leiden, The Netherlands; J.Corver@lumc.nl; 4 Center for Infectious Diseases (LU-CID), Leiden University, 2333 ZA Leiden, The Netherlands; 5 Centre for Microbial Cell Biology, Leiden University, 2333 ZA Leiden, The Netherlands; 6 Department of Public Health, University of California, Irvine, CA 92697, USA; danica.resu@gmail.com; 7 Center for Proteomics and Metabolomics, Leiden University Medical Center, Leiden University, 2333 ZA Leiden, The Netherlands; P.J.Hensbergen@lumc.nl; 8 Department of Molecular Biology & Biochemistry, University of California, Irvine, CA 92697, USA

**Keywords:** *Clostridioides difficile*, NanoLuc, point-of-care device, bioluminescent sensor, protease sensor, PPEP-1

## Abstract

Current assays for *Clostridioides difficile* in nonhospital settings are outsourced and time-intensive, resulting in both delayed diagnosis and quarantining of infected individuals. We designed a more rapid point-of-care assay featuring a “turn-on” bioluminescent readout of a *C. difficile*-specific protease, PPEP-1. NanoLuc, a bright and stable luciferase, was “caged” with a PPEP-1-responsive peptide tail that inhibited luminescence. Upon proteolytic cleavage, the peptide was released and NanoLuc activity was restored, providing a visible readout. The bioluminescent sensor detected PPEP-1 concentrations as low as 10 nM. Sensor uncaging was achieved within minutes, and signal was captured using a digital camera. Importantly, the sensor was also functional at ambient temperature and compatible with fecal material, suggesting that it can be readily deployed in a variety of settings.

## 1. Introduction

*Clostridioides difficile*, a widespread anaerobic bacterium, is the leading cause of infectious nosocomial diarrhea [1,2]. *C. difficile* infection (CDI) can further lead to severe pathologies, including pseudomembranous colitis and toxic megacolon [1,3]. CDI is highly prevalent in hospitals and long-term care facilities (LTCFs), particularly among patients of advanced age [4]. Infection of the elderly is further exacerbated by the overprescription of broad-spectrum antibiotics [5]. Antibiotic treatment disrupts the normal intestinal flora, leading to increased risk of CDI [6]. Rapid diagnosis followed by proper treatment or isolation of patients can greatly reduce *C. difficile* spread among vulnerable individuals in a densely populated setting [7].

Current CDI diagnoses rely on the detection of two well-studied exotoxins—toxin A (TcdA) and toxin B (TcdB) [8]. These proteins are part of a larger family of toxins secreted by clostridial bacteria [9]. Thus, TcdA and TcdB are not completely unique to *C. difficile*. Other proteins secreted by the pathogen could potentially be used for more accurate diagnoses [10,11]. Included in this group is a unique zinc metalloprotease, PPEP-1, that is conserved among *C. difficile* strains and thought to be necessary for pathogen mobility and colonization of the gut [10,11]. PPEP-1 belongs to a family of proteases with a specific preference for cleaving Pro–Pro bonds in an overall proline-rich motif [11,12]. The proline residue in the P1’ position (VNP/PVPP, scissile bond denoted by slash) is not accommodated by other proteolytic enzymes, including trypsin. Moreover, the consensus sequence of PPEP-1 is distinct from other PPEP proteases [11,12]. Thus, the unique cleavage activity of PPEP-1, which to our knowledge is exclusive to *C. difficile*, could serve as a highly specific and novel readout for CDI.

Standard CDI detection relies on the incubation of patient stool samples with TcdA- and TcdB-sensitive cell lines [8]. This approach is technically demanding and time-intensive, requiring ~1–5 days to diagnose from the time of collection. Multistep protocols combining TcdA/TcdB immunoassays with PCR verification have been developed but are limited in sensitivity and still require several days to process [13,14]. Conventional CDI tests further require trained technicians and laboratory equipment, both of which are lacking in LTCFs. Consequently, CDI testing is typically outsourced, leading to delays in diagnoses and increased risk of rampant infection. Thus, there remains a need for point-of-care (POC) CDI detection to better guide patient isolation and treatment.

An ideal POC platform would be specific and sensitive to *C. difficile* and also easily interpretable. The readout would require minimal technical skill and equipment. The reagents would also be amenable to long-term storage in noncontrolled settings. Many of these criteria are fulfilled by bioluminescent sensors. Bioluminescence involves visible light production from small molecule luciferins and luciferase enzymes. NanoLuc [15] (Nluc), one of the brightest and most stable luciferases, has been used in sensing platforms for a variety of target biomolecules. One popular method leverages resonance energy transfer (RET) between Nluc and fluorophore acceptors. In the presence of analyte, the sensors exhibit a change in emission profile; such shifts have been used to detect therapeutic drugs [16,17], metabolites [18,19,20], and antibodies [21]. Some Nluc assays have even been deployed on paper in noncontrolled environments [16,18]. These and other RET sensors rely on a post-image processing step to ratio the outputs from the luciferase and fluorophore components.

Intensiometric sensors that provide binary “on–off” readouts can be operationally simpler for untrained individuals. Luciferase-based intensiometric sensors have been used to detect small molecules and various biomolecules [22,23]. Many involve circularly permutated or split variants of the light-emitting enzyme [22,23,24]. For example, a circularly permuted version of firefly luciferase was engineered for protease detection [22]. However, the low photon output of the enzyme precludes its application as a POC sensor. We surmised that the increased thermostability and luminescence of Nluc would be more amenable for POC designs. Coupling PPEP-1 activity with a “turn-on” Nluc sensor could quickly inform on CDI, without the need for expensive equipment.

## 2. Materials and Methods

### 2.1. General Bioluminescence Imaging

All analyses were performed in black 96-well plates (Greiner Bio-One, Monroe, NC, USA). Plates containing luminescent reagents were imaged in a dark, light-proof chamber using an IVIS Lumina (PerkinElmer, Waltham, MA, USA) CCD camera chilled to −90 °C. The stage was kept at 37 °C during imaging and the camera was controlled using Living Image software. Exposure times were set to 1 s and binning levels were set to medium. Regions of interest were selected for quantification and total flux values were analyzed using Living Image software. All data were exported to Microsoft Excel or PRISM (GraphPad, San Diego, CA, USA) for further analysis.

### 2.2. Digital Camera Bioluminescence Imaging

All analyses were performed in black 96-well plates. Samples were imaged 1 min after furimazine (Promega, Madison, WI, USA) addition in a polystyrene icebox to exclude ambient light. Pictures were captured using a OnePlus 8 Pro (Shenzhen, China) cellular phone with exposure time of 8 s, ISO value of 3200.

### 2.3. General CgNBit PPEP-1 Luminescence Assay

Assays were performed in black 96-well plates in 50 mM sodium phosphate buffer (pH 7.4). For each sample, 10 nM sensor was incubated for 30 min at 37 °C in the presence or absence of 100 nM PPEP-1. Furimazine (2 µL of commercial stock) was then added and the plate was immediately imaged as described above.

### 2.4. General CgNluc PPEP-1 Luminescence Assay

Assays were performed in 96-well plates in phosphate-buffered saline (PBS, 137 mM NaCl, 2.7 mM KCl, 10 mM Na_2_HPO_4_, 1.8 mM KH_2_PO_4_). For each sample, 1 µM sensor was incubated for 30 min at 37 °C in the presence or absence of PPEP-1. Samples were then serially diluted to a final concentration of 1 nM sensor. Furimazine (2 µL of commercial stock) was then added and the plate was imaged 1 min later as described above.

### 2.5. Liquid Chromatography–Mass Spectrometry

Samples were analyzed on a Waters Xevo G2-XS QTOF Mass Spectrometer (Milford, MA, USA). For each sample, injection volumes (10 µL of a 1 µM solution) were analyzed after in-line desalting on a phenyl guard column. Waters MassLynx software’s MaxEnt1 deconvolution was used to process the acquired ESI mass spectra.

### 2.6. Clostridioides difficile Culture

*C. difficile* strain 630Δ*erm* was grown anaerobically in an anaerobic cabinet (Don Whitley A55, Bingley, UK) at 37 °C in minimal medium broth [25]. Cells were pelleted 48 h post-inoculation and the supernatant was collected. Subsequently, the supernatant was sterilized through a 0.2 µm filter and stored at −20 °C. GMO permit number, IG 07-053.

## 3. Results

### 3.1. Proteolytic Sensor Design

**Figure 1:**
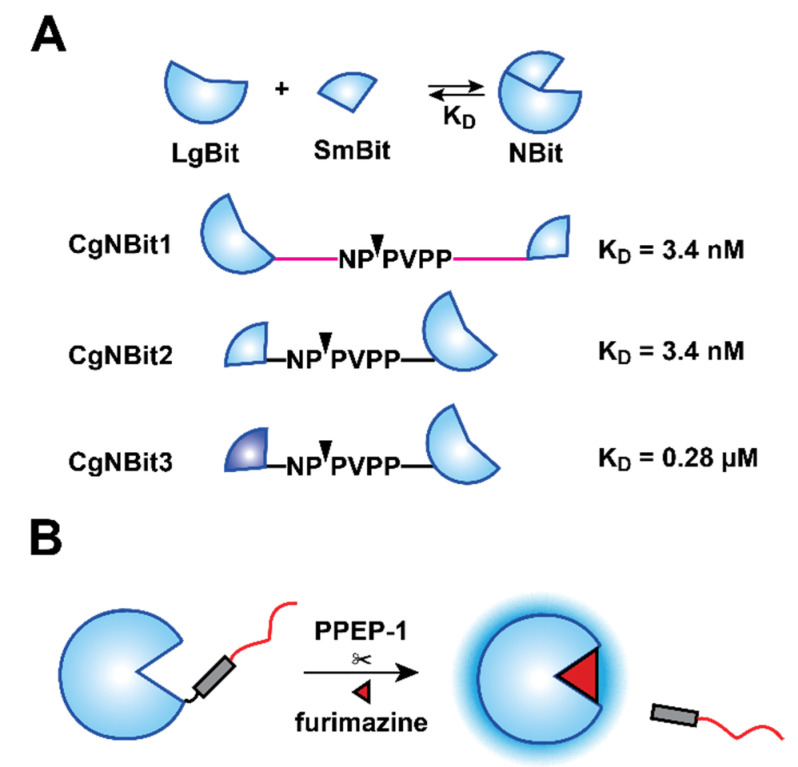
PPEP-1 bioluminescent sensor designs. (**A**) Sensor designs based on NanoBiT. LgBiT and SmBit fragments were separated via linkers comprising PPEP-1 cleavage motifs. *K*_D_ values denote the SmBiT peptide sequence used in each design. Scissile bonds are denoted by triangles. (**B**) Sensor design based on a caged version of Nluc. A caging peptide was fused to the C-terminus of Nluc via a PPEP-1 cleavage sequence. PPEP-1 releases the cage, turning on Nluc activity. Light is thus produced in the presence of furimazine.

We initially envisioned using a split variant of Nluc, the NanoBiT (NBiT) system, for sensor development (Figure 1A). NBiT comprises two pieces, a small peptide (SmBit, 1.3 kDa) and a larger fragment (LgBiT, 18 kDa) [24]. The components are “dark” on their own, but when bound, functional Nluc is formed and robust light emission is observed. Thus, the split reporter can provide a simple “on–off” response. NBiT has been used for imaging a variety of protein–protein interactions involving phosphatases [26], G proteins [27], and other biomolecules [28]. More recent work from Baker et al. combined NBiT with a de novo designed protein switch to detect various analytes [23]. This sensor features a structurally perturbed SmBiT that cannot reassemble with LgBiT in the absence of target analyte. In the presence of analyte, though, functional SmBiT is released and NBiT complementation ensues.

We aimed to use a similar strategy to perturb intramolecular NBiT complementation and make it responsive to *C. difficile*. Our initial designs separated SmBiT and LgBiT via linkers comprising PPEP-1 cleavage motifs (Figure 1A). When short linkers were used, intramolecular complementation was observed regardless of protease activity. Increased separation improved PPEP-1-dependent signal, but the readouts were still suboptimal (Figure S1A,B, Supplementary Materials). Studies from Baker et al. suggested that blocking unproductive intermolecular associations could further improve signal-to-noise ratios [23]. We tested this design strategy using coiled-coil domains to obstruct off-target interactions [29]. Unfortunately, this sensor also exhibited high levels of background complementation in the absence of PPEP-1 (Figure S1C).

Given the difficulties encountered with the split reporter, we examined whether native Nluc activity could be inhibited, then restored, via PPEP-1 activity. Although a crystal structure of Nluc bound with its substrate (furimazine, FRZ) has not yet been reported, the active site of Nluc is thought to comprise a surface-exposed pocket near the C-terminus [30,31]. We performed additional docking studies to corroborate this prediction (Figure S2). We hypothesized that adding an unstructured element, such as a peptide tail, could sufficiently block substrate access to the active site and impede light emission. To test this idea, we fused a variety of common linker motifs [32] to the C-terminus of Nluc and expressed the constructs in HEK293T cells. Among the panel tested, a repeating unit of glycine and serine residues (GGGGSGGGGS; G_4_S_x2_) inhibited Nluc luminescence to the greatest extent (~10-fold, Figure S3). It is interesting to note that Nluc is most commonly fused to other proteins at its N-terminus, likely minimizing disruption to the luciferin binding pocket [33,34]. Functional C-terminal fusions of Nluc have been reported, but they comprise alternative glycine–serine motifs or much shorter linkers [35,36].

**Figure 2:**
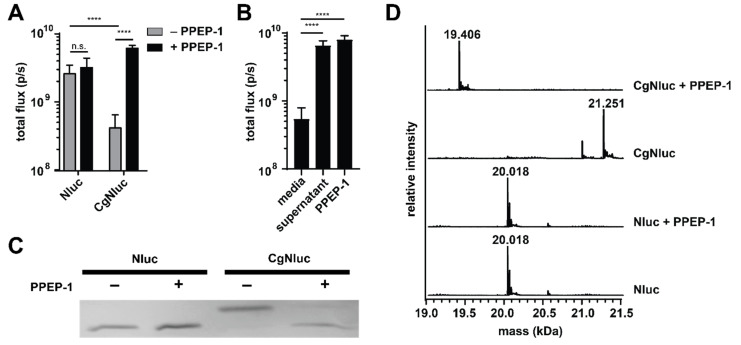
PPEP-1-dependent uncaging of CgNluc. (**A**) Nluc and CgNluc (1 µM) were treated with recombinant PPEP-1 (1 µM, +) or media alone (−) for 30 min. Samples were diluted to 1 nM and imaged with furimazine. (**B**) CgNluc was incubated with culture media, *C. difficile* supernatant, or 1 µM recombinant PPEP-1 prior to imaging. (**C**) SDS-PAGE analysis of Nluc and CgNluc in the presence (+) or absence (−) of 1 µM PPEP-1. (**D**) LC-MS analysis of Nluc and CgNluc in the presence or absence of PPEP-1. **** *p* < 0.0001; n.s., nonsignificant.

To develop a protease-responsive caged Nluc (CgNluc) biosensor, we incorporated the PPEP-1 cleavage motif between Nluc and the G_4_S_x2_ peptide (Figure 1B). A His_6_ purification tag was also included in the C-terminal tail. The inhibitory motif would be released in the presence of PPEP-1, thereby restoring the activity of Nluc and light emission. To test this strategy, CgNluc was expressed and purified by affinity chromatography. The sensor exhibited diminished activity in vitro, emitting ~7-fold fewer photons than Nluc itself. In the presence of 1 μM PPEP-1 and furimazine, an 18.5-fold increase in luminescence was observed. By contrast, Nluc emission was unaffected by PPEP-1 (Figure 2A and Figure S4A). CgNluc also exhibited a 14-fold increase in light output when incubated in supernatant from *C. difficile* (Figure 2B and Figure S4B). The level of signal induction was similar to that achieved with 1 µM recombinant protease. SDS-PAGE analysis confirmed that CgNluc was cleaved by PPEP-1 (Figure 2C). LC-MS analysis further confirmed that the peptide “cage” of CgNluc (PVPPGGGGSGGGGSHHHHHH, 1.86 kDa) was removed (Figure 2D). No cleavage products were observed when Nluc itself was treated with PPEP-1.

### 3.2. Sensor Optimization and Characterization

**Figure 3:**
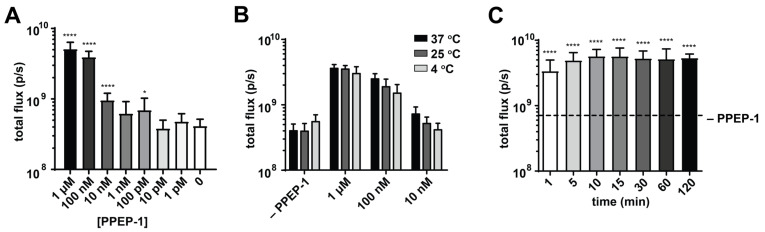
Optimization of the sensor readout. CgNluc (1 µM) was incubated with varying concentrations of PPEP-1 for 30 min (**A**) at 37 °C or (**B**) different temperatures. (**C**) CgNluc (1 µM) was incubated with PPEP-1 for various time periods prior to imaging. All samples were diluted to 1 nM for imaging. **** *p* < 0.0001; * *p* < 0.05.

Next, we tested assay conditions to optimize the sensitivity and dynamic range of the readout. Sensor uncaging and subsequent turn-on relies on the enzymatic activity of both the protease and the liberated luciferase. Both enzymes can be impacted by several factors including the assay buffer, incubation time, and temperature. To determine the basal sensitivity of CgNluc to PPEP-1, the sensor was incubated with titrating concentrations of the protease for 30 min at 37 °C. Increased bioluminescent outputs were observed with increasing concentrations of PPEP-1 (Figure 3A), with an ~10-fold increase in signal at the highest dose. Lower concentrations of protease (e.g., 10 nM PPEP-1) resulted in partial cleavage of the sensor, but still detectable changes in luminescence (Figure S5A). In all cases, cleavage products were confirmed by LC-MS analysis (Figure S6). The uncaging assay was also examined in a variety of buffers. Signal turn-on was most pronounced in PBS (Figure S5B).

POC biosensors must be stable over a range of temperatures due to their deployment in noncontrolled settings. Although Nluc has been shown to be exceedingly thermal stable [15], the addition of a caging motif could affect its overall stability and diminish sensor sensitivity. Application at ambient temperature, in particular, is beneficial in LTCFs that typically have little to no equipment. To examine this possibility, we incubated CgNluc in the presence of titrating amounts of PPEP-1 at room temperature (25 °C) for 30 min. Sensor activation and sensitivity were similar to those achieved at 37 °C (Figure 3B and Figure S5C). The sensor was also functional at 4 °C, although slightly less responsive likely due to decreased PPEP-1 activity [15]. Additionally, CgNluc samples stored in 50% glycerol at –20 °C for 4 months performed comparably to fresh batches (Figure S7A,B). These findings reinforce the feasibility of a luciferase-based POC device, as the assay can be performed in nonlaboratory settings. The reagents are also amenable to long-term storage.

Along with buffer and temperature, we investigated the impact of incubation time on sensor response. PPEP-1 (0.5 µg) has been previously shown to cleave its natural substrate (3 µg) within minutes of incubation [11]. We also confirmed that CgNluc uncaging occurs rapidly in the presence of the protease (1 μM), providing a robust induction in photon emission (Figure 3C and Figure S5D). Signal turn-on was observed after only one minute of incubation, with maximal signal intensity achieved after 5–10 min. Such rapid readouts are desirable in LTCFs, to quickly inform on whether further patient testing or treatment is needed.

### 3.3. Digital Camera Imaging of CgNluc

**Figure 4:**
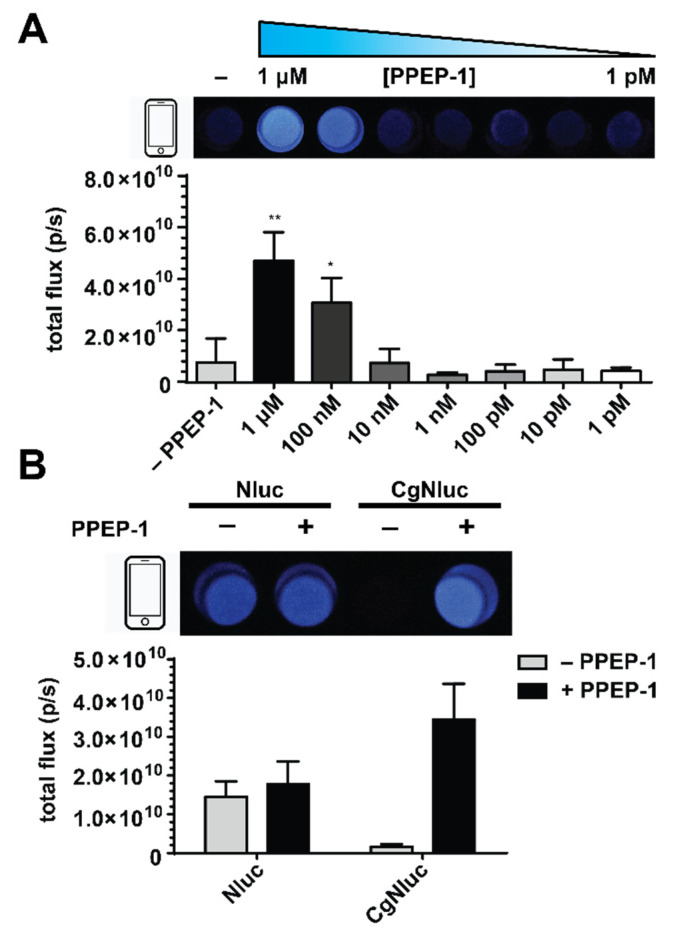
Digital camera detection of PPEP-1 with CgNluc biosensor. (**A**) (top) Images from CgNluc (1 µM) incubated with titrating amounts of PPEP-1. (bottom) Quantified flux values are shown. (**B**) (top) Images from Nluc and CgNluc (1 µM) treated with PPEP-1 (1 µM, +) or media alone (−) for 30 min. (bottom) Quantified flux values are shown. For (**A**,**B**), samples were diluted to 10 nM and imaged with a phone camera. ** *p* < 0.01; * *p* < 0.05.

Another key requirement for POC assays is an easily interpretable readout—ideally, a binary “on–off” signal. Standard digital cameras are useful in this context and capable of capturing Nluc emission [18,21,37,38]. To examine the feasibility of CgNluc imaging with a digital camera, we first established an optimal concentration of the sensor (10 nM) for facile detection (Figure S8). Solutions of the sensor were then treated with varying concentrations of PPEP-1. Distinct signal was observed in the presence of >100 nM protease (Figure 4A). No signal was observed in the absence of PPEP-1, even with 8 s of exposure. Comparable concentrations of native Nluc were unaffected by the protease (Figure 4B).

**Figure 5:**
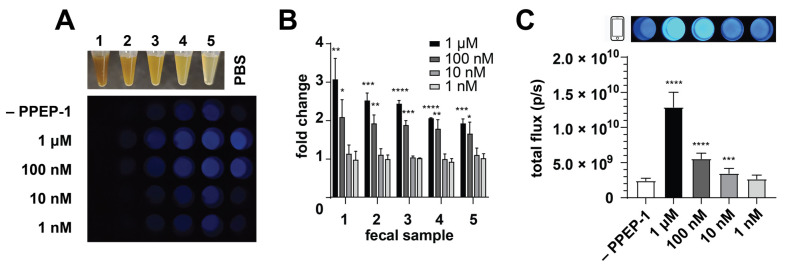
Biosensor performance in fecal samples. (**A**) Representative phone camera images of CgNluc (1 µM) incubated with various samples of feces and PPEP-1. Serial dilutions (1:2) were performed using PBS. Samples were diluted to 10 nM CgNluc and imaged. (**B**) Bioluminescent outputs of CgNluc (1 µM) treated with PPEP-1 (0–1 μM), then added to fecal samples to a final concentration of 10 nM CgNluc and quantified. (**C**) CgNluc (1 µM) incubated directly in fecal samples with titrating concentrations of PPEP-1. Representative phone camera images at 10 nM CgNluc are shown above. Samples were diluted to 1 nM CgNluc for quantification. **** *p* < 0.0001; *** *p* < 0.001; ** *p* < 0.01; * *p* < 0.05.

Bioluminescence is advantageous for sensitive imaging directly in heterogeneous environments. We thus surmised that CgNluc could be used directly in fecal material for CDI detection, with minimal to no sample preparation. Direct addition would mitigate the need for filtering and other sample clarification steps (associated with current CDI assays) that require technician handling. Rodent feces were used as a proxy for human samples. The feces were resuspended at various dilutions in PBS to examine the impacts on bioluminescent detection. CgNluc was preincubated with titrating concentrations of PPEP-1 and then added to the fecal samples. High turbidity reduced sensor visibility, but CgNluc could be robustly detected in 4-fold diluted fecal samples (Figure 5A). The fecal matter also diminished the sensor’s dynamic range, with an ~2-fold increase in signal observed when uncaged with 100 nM PPEP-1 (Figure 5B). The reduced signal was attributed to increased background luminescence in the heterogeneous sample. Similar increases in background were observed in media comprising high concentrations of protein (Figure S9).

To evaluate the sensitivity of CgNluc for CDI detection in stool, the sensor was directly added to fecal samples along with recombinant PPEP-1. The samples were diluted to a final concentration of 10 nM CgNluc for imaging. This final dilution step mitigates the reduction in bioluminescent signal from fecal turbidity, similar to a wash step. Upon dilution, CgNluc enabled sensitive detection of 100 nM PPEP-1 in fecal samples (Figure 5C). The signal turn-ons observed with 1 µM and 100 nM protease were 5.5-fold and 2.3-fold, respectively (Figure S10). Both samples could be readily detected on a cell phone camera. These results suggest that CgNluc can potentially be applied in a direct sampling assay, with additional optimization. For example, conjugation of the sensor to a solid support could enable a true wash step, improving the overall dynamic range.

## 4. Discussion

In this work, we developed a novel protease-dependent bioluminescent sensor for *C. difficile* detection. Our initial designs based on split NanoBiT assembly were unsuccessful, primarily due to competing intermolecular complementation. Such undesired binding events could potentially be inhibited with additional sensor optimization [23]. However, additional modeling and structural modifications would likely be required. We thus designed an alternative sensor based on a caged version of Nluc. This probe is responsive to PPEP-1, a *C. difficile*-specific protease. Readouts can be achieved with a digital camera within minutes of incubation at ambient temperature. Furthermore, the luciferase provides sufficiently bright signal, even in highly turbid fecal samples. This characteristic suggests the feasibility of developing a POC device for direct stool sampling.

While more sensitive assays for CDI are available, sample processing typically requires laboratory equipment and skilled technicians. These limitations delay diagnoses and can greatly increase the possibility of rampant *C. difficile* infection in areas with poor accessibility to standard detection methods. CgNluc could be advantageous in such settings. Although PPEP-1 may not be diagnostic of toxic *C. difficile*, it can serve as a biomarker for rapid identification of the pathogen. Preliminary readouts on suspected cases could be achieved quickly, allowing for preemptive isolation of patients while more sensitive—but time-intensive—assays confirm the diagnoses.

While CgNluc is sensitive to PPEP-1, we do not yet fully understand the role of the inhibitory peptide in the sensor design. Future applications for CDI detection would benefit from additional engineering. Further modification of the caging motif could potentially improve enzyme inhibition and increase the dynamic range within a wider range of complex environments. Immobilization of the sensor to a solid support would also provide a more user-friendly and deployable assay. This format could also improve the sensitivity of the assay by allowing for more rigorous washing steps. Importantly, CgNluc also serves as a blueprint for additional biosensor designs. The proteolytic cleavage sequence is potentially modular and could be made responsive to other proteases. A series of CgNluc probes could be used to rapidly detect other pathogens or disease markers.

## Data Availability

The data presented in this study are available within the article and associated supplementary material.

## Supplementary Materials

The following are available online at https://www.mdpi.com/article/10.3390/s21227485/s1, Figure S1: NanoBiT sensor designs, Figure S2: Docking analysis of furimazine with Nluc, Figure S3: Effect of C-terminal linkers on Nluc activity, Figure S4: Fold-improvement of sensor in presence of PPEP-1, Figure S5: Optimization of sensor readout, Figure S6: LC-MS of CgNluc cleavage, Figure S7: Sensor viability after prolonged storage, Figure S8: Digital imaging with CgNluc, Figure S9: Nluc intensity in the presence of high protein concentration, Figure S10: CgNluc performance in fecal material, Table S1: Primers used in plasmid construction, Supplementary Methods.
