# Supplementary material for: A Bioluminescent Sensor for Rapid Detection of PPEP-1, a Clostridioides difficile Biomarker
Source: Sensors (Basel). 2021 Nov 11;21(22):7485. doi: 10.3390/s21227485 (PMC8624784; doi:10.3390/s21227485)
Supplement: Supplementary file 1 [file sensors-21-07485-s001.zip › sensors-1418868-supplementary.pdf]

Supplementary Material

A bioluminescent sensor for rapid detection of PPEP-1, a *Clostridioides difficile* biomarker

Kevin K. Ng[1], Zachary Reinert[2], Jeroen Corver[5], Danica Resurreccion[4], Paul J. Hensbergen[6], Jennifer Prescher[1,2,3,*]

[1] Department of Pharmaceutical Sciences, University of California, Irvine, California, 92697, United States
[2] Department of Chemistry, University of California, Irvine, California, 92697, United States
[3] Department of Molecular Biology & Biochemistry, University of California, Irvine, California, 92697, United States
[4] Department of Public Health, University of California, Irvine, California, 92697, United States
[5] Department of Medical Microbiology, Section Experimental Bacteriology Center of Infectious Diseases, Leiden University, Leiden University Medical Center, Leiden, the Netherlands
[6] Center for Proteomics and Metabolomics, Leiden University, Leiden University Medical Center, Leiden, the Netherlands

*Email: jpresche@uci.edu

Contents

Supplementary Materials and Methods 2
- Plasmid construction 2
- Protein expression and purification 2
- Mammalian cell culture 2
- Flow cytometry 2
- Bioluminescence imaging with mammalian cells 3
- NanoLuc docking analysis 3

Supporting Figures 4
- Figure S1 4
- Figure S2 5
- Figure S3 5
- Figure S4 6
- Figure S5 6
- Figure S6 7
- Figure S7 8
- Figure S8 8
- Figure S9 9
- Figure S10 9

References 9

Table S1. Primers used in plasmid construction. 10

Supplementary Materials and Methods

Plasmid construction

NBiT-based sensor plasmids and PPEP-1 were constructed using overlapping circular polymerase extension cloning (CPEC) [1]. Relevant sensor gene fragments (LgBiT and SmBiT) were inserted into pCold or pET vectors. PPEP-1 was inserted into a pET vector. Vectors were amplified using the following primers:

pCold
5’ – TAAGAATTCAAGCTTGTCGACCTGC – 3’ and
5’ – CATCATATGCCTACCTTCGATATGATG -3’

pET
5’ – CATCACCATCACCATCACTAAATAGAATTCTAATAATCCGGCTGCTAACAAAG – 3’ and
5’ – TTTGTTTAACTTTAAGAAGGAGATATActcgag – 3’

CgNluc and Nluc-linker plasmids were constructed through Gibson assembly [2]. Relevant gene fragments were amplified and inserted into pET or pcDNA vectors. CgNluc was inserted into a pET vector. Nluc-linker gene fragments were inserted into pcDNA vectors. Linearized vectors were generated via digestion with restriction enzymes (New England BioLabs). pET vector was digested with *Xho*I and *EcoR*I. pcDNA vector was digested with *Hind*III and *EcoR*I.

Primers for all corresponding plasmids can be found in Table S1 below.

Protein expression and purification

CgNBiT-1 through -3 and Invasin-NBiT were encoded in a pCold vector. CgNluc, CgNBiT, and PPEP-1 were encoded in a pET vector. Sensors and proteins were expressed in *E. coli* BL21 cells using 1 L of LB medium. Expression was induced at an optical density (OD600) of ~0.6 by addition of 0.5 mM isopropyl-β-D-thiogalactopyranoside (IPTG), followed by 4 h of incubation at 37 °C. Sensors encoded in pCold vectors were cold-shocked on ice for 20 min prior to induction. Cells were harvested by centrifugation (4,000x*g*, 10 min, 4 °C). Cells were resuspended in lysis buffer (30 mL, 50 mM Tris HCl, 150 mM NaCl, 0.5% Tween-20, 1 mM phenylmethylsulfonyl fluoride, pH 7.4). Cells were sonicated (QSonica) at 40% amplitude, using 2 sec on/2 sec off intervals for 15 min. Cell debris was removed through centrifugation (10,000x*g*, 1 h, 4 °C). Proteins were purified by Ni-NTA affinity chromatography. Columns were washed with wash buffer (20 mM imidazole, 50 mM sodium phosphate, pH 7.4). Proteins were eluted from columns with elution buffer (200 mM imidazole, 50 mM sodium phosphate, pH 7.4). Protein was dialyzed overnight at 4 °C into phosphate buffer (50 mM sodium phosphate, pH 7.4). Protein was concentrated to ~500 mL using Amicon Ultra-15 Centrifugal Filter Units (Merck Millipore MWCO 3 kDa). Protein concentrations were determined using a JASCO V730 UV-vis spectrophotometer (280 nm). SDS-PAGE analyses were performed to verify purity, and gels were stained with Coomassie R-250.

Mammalian cell culture

HEK293T cells were cultured in DMEM (Corning) supplemented with 10% (vol/vol) fetal bovine serum (FBS, Life Technologies) penicillin (100 U/mL), and streptomycin (100 μg/mL). Cells were maintained in a 5% CO_2 water-saturated incubator at 37 °C. Transient transfections of plasmid DNA were performed using cationic lipid formulations (Lipofectamine 2000; Invitrogen). Transfection efficiencies were analyzed via eGFP expression and measured 24 h post-transfection via flow cytometry.

Flow cytometry

Cells were trypsinized and washed twice in PBS prior to analysis on a ACEA NovoCyte Flow Cytometer. For each sample, 10,000 live cell events were collected, and data were analyzed using NovoExpress software (version 1.3.0).

Bioluminescence imaging with mammalian cells

HEK293T cells were plated in 12-well cell culture plates (5.0 x 10^6 per well) and transfected with Nluc–linker constructs after 24 h (1 µg DNA, 1 µL Lipofectamine 2000). Cells were trypsinized and re-seeded into black 96-well plates. Furimazine (2 µL) was added to each sample and cells imaged as described above. Cells were also analyzed via flow cytometry to quantify transfection efficiency based on GFP signal.

NanoLuc docking analysis

Furimazine was docked into the crystal structure of NanoLuc (PDB:5IBO) using AutoDock Vina [3]. Protein structures were converted from pdb into pdbqt format using MGL Tools [4]. The structure of NanoLuc was fixed during docking and single bonds in furimazine were allowed to rotate. The grid box was constructed to fully cover the entire protein. The 9 lowest energy conformations were imported into PyMol and visually inspected.

Supporting Figures

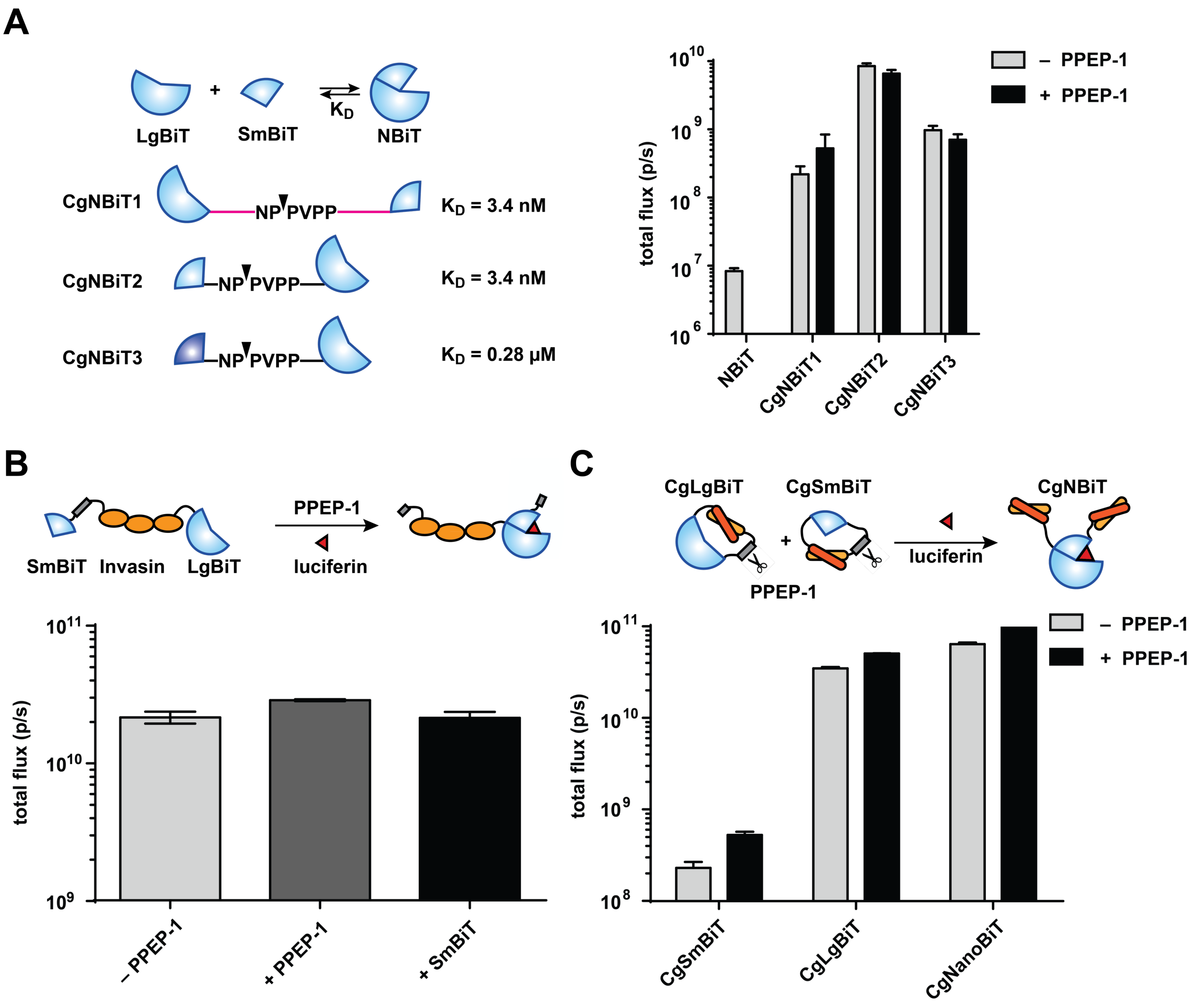

Figure S1. NanoBit sensor designs. (A) (left) LgBiT and SmBit fragments were separated using various linker lengths. K_D values denote the SmBiT peptide sequence used in each design. Scissile bonds are denoted by triangles. (right) Bioluminescent output from CgNBiT sensors in the presence (+) or absence (–) of PPEP-1 (1 μM). (B) LgBiT and SmBit fragments were separated by 3 repeats of invasin. Bioluminescent outputs from the sensor in the presence (+) or absence (–) of PPEP-1 (1 μM) are shown below. Exogenous SmBiT peptide (5 μM) was also included as a control. (C) LgBiT and SmBit fragments were individually "caged" with E/K coiled-coil motifs (red, orange). Bioluminescent outputs from each fragment alone or together in the presence (+) or absence (–) of PPEP-1 (1 μM) are shown below.

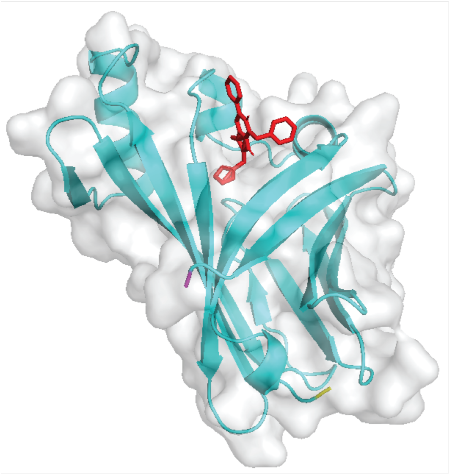

Figure S2. Docking analysis of furimazine with Nluc (performed with AutoDock Vina). Furimazine is shown in red and the structure of Nluc is labeled in cyan. The N-terminus is colored yellow and the C-terminus is colored pink.

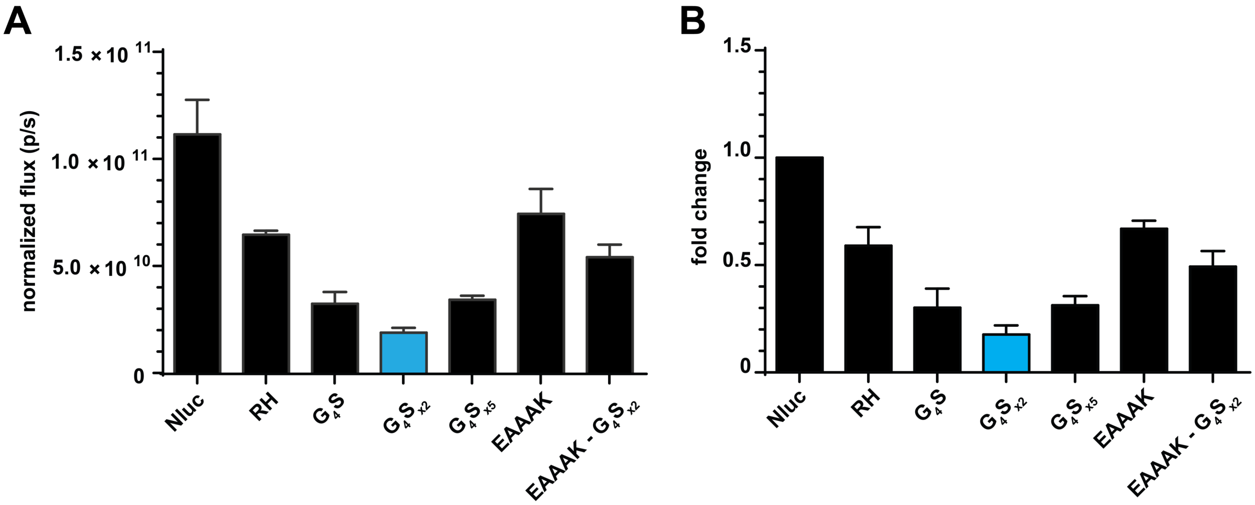

Figure S3*.* Effect of C-terminal linkers on Nluc activity. (A) Bioluminescent outputs of HEK293T cells expressing various Nluc conjugates. Flux values (normalized for overall expression) are shown. (B) Fold-change in light output of Nluc conjugates compared to native Nluc.

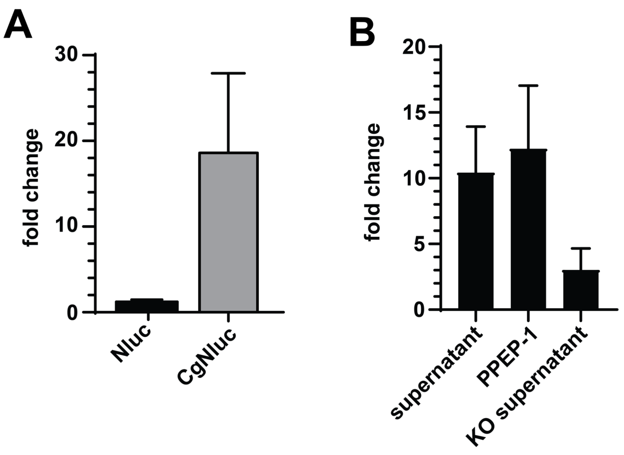

Figure S4. Fold improvement of sensor in the presence of PPEP-1. Data from Figures 2A and 2B plotted as (A) fold-change in luminescence of Nluc (1 µM) or CgNluc (1 µM) treated with 1 µM PPEP-1 compared to Nluc alone (B) fold-change in luminescence of CgNluc (1 µM) incubated with wild-type *C. difficile* supernatant, supernatant from PPEP-1 knockout *C. difficile*, or recombinant PPEP-1 (1 µM) compared to CgNluc in culture media. All samples were diluted to 1 nM for imaging.

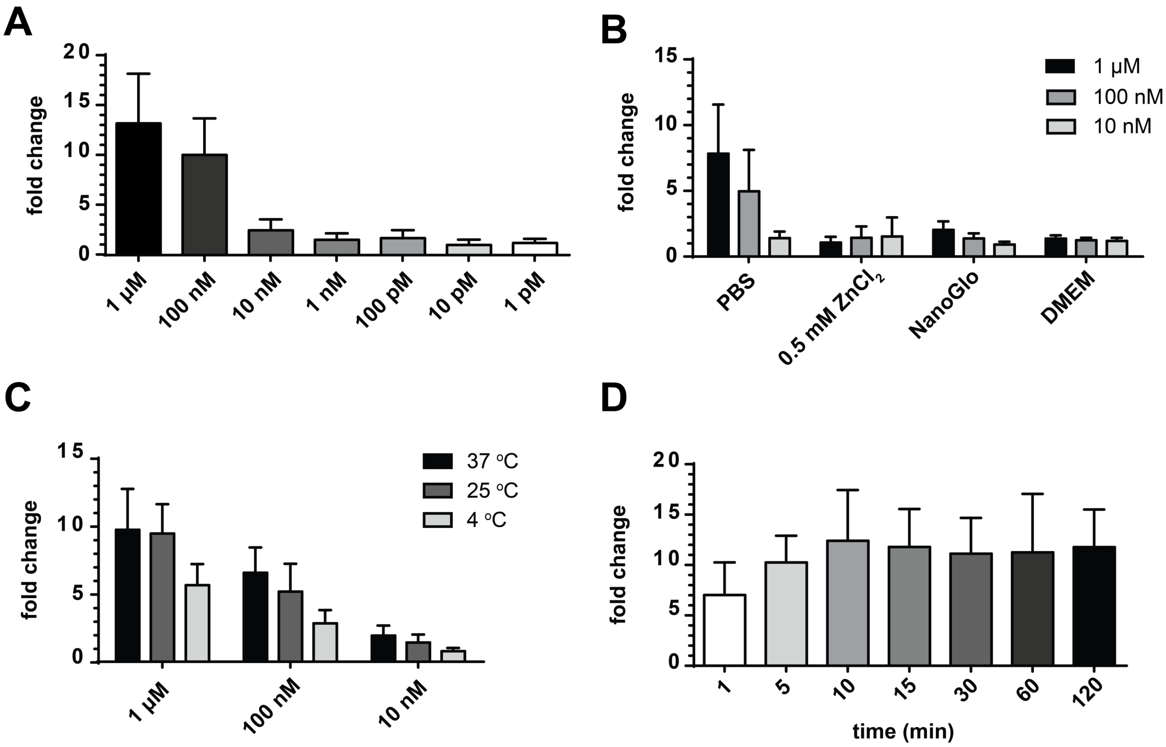

Figure S5 Optimization of sensor readout. Data from Figure 3 plotted as the fold-change in luminescence for each condition compared to signal in the absence of PPEP-1. CgNluc (1 µM) was incubated (A) with varying concentrations of PPEP-1 for 30 min at 37 °C, (B) in different buffers, or (C) at different temperatures. (D) CgNluc (1 µM) was incubated with PPEP-1 over time prior to imaging. All samples were diluted to 1 nM for imaging.

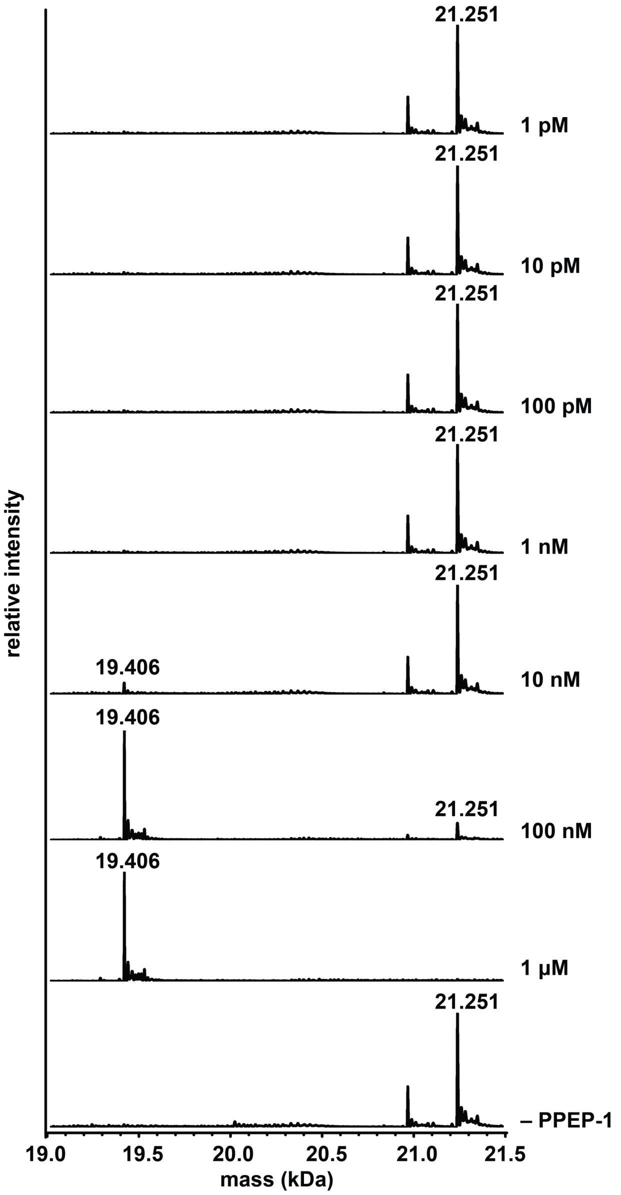

Figure S6. Cleavage analysis of CgNluc with titrating concentrations of PPEP-1. CgNluc (1 µM) was incubated with PPEP-1 (0-1 µM) for 30 min at 37 °C and analyzed via LC-MS.

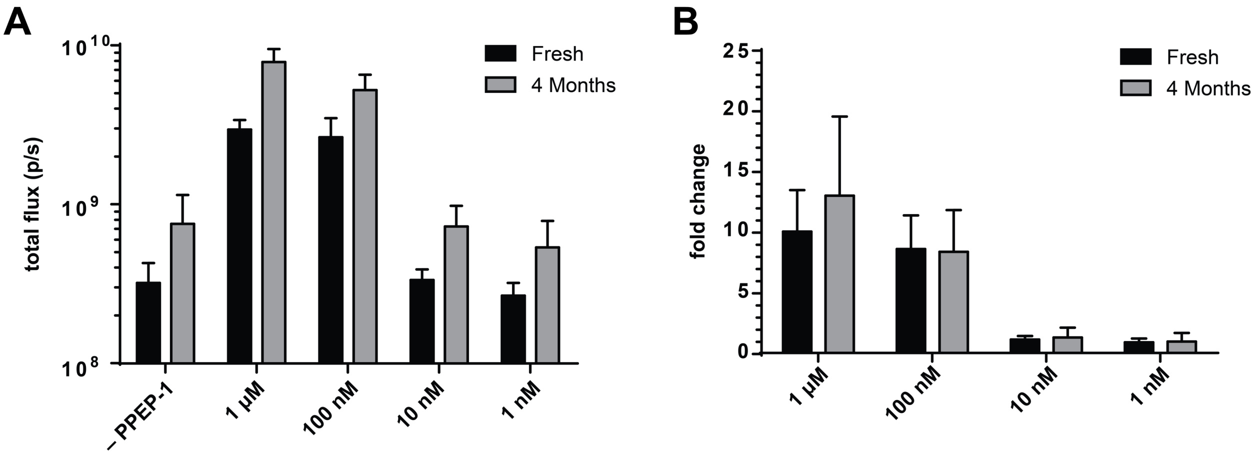

Figure S7. Sensor viability after prolonged storage. (A) CgNluc stocks stored for 4 months (at -20 °C in 50% glycerol) were tested alongside freshly prepared stocks. CgNluc samples (1 µM) were incubated with varying amounts of PPEP-1 (0-1 µM). Samples were diluted to 1 nM for imaging. (B) Fold-change in total flux from stored stocks compared to freshly prepared stocks in (A).

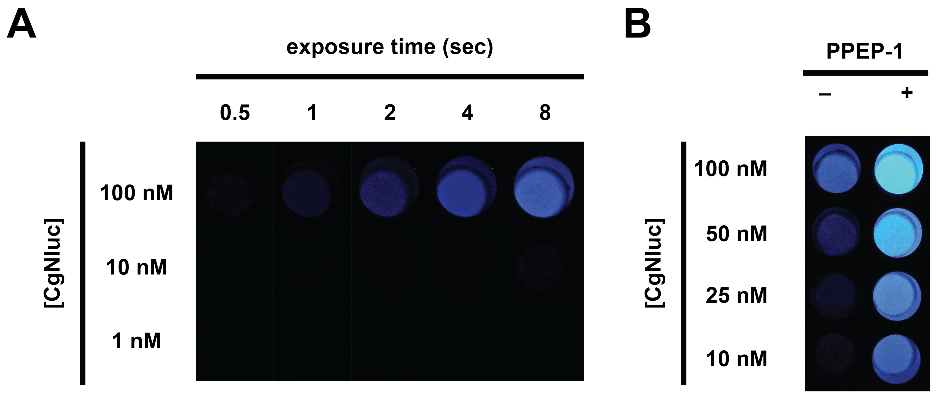

Figure S8. Digital imaging with CgNluc. (A) Digital camera images from titrating concentrations of CgNluc and varying exposure times. (B) Images from CgNluc incubated with (+) or without (–) of PPEP-1 (1 µM), using an 8 s exposure.

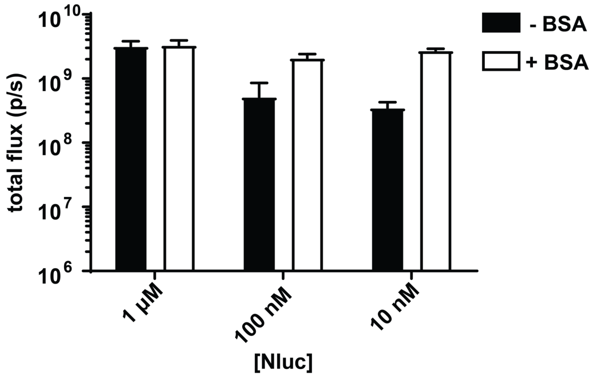

Figure S9. Nluc intensity in the presence of high protein concentration. Titrating concentrations of Nluc incubated in the absence (–) or presence (+) of 1 µM BSA. Samples were incubated for 30 min at 37 °C.

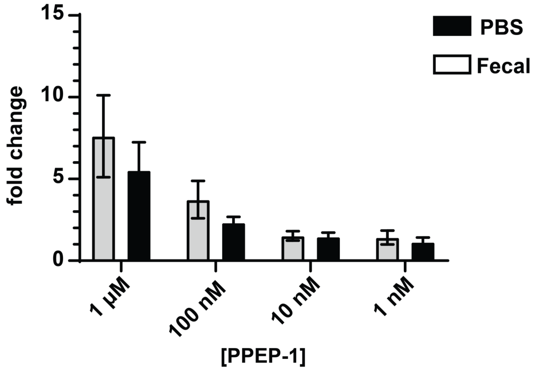

Figure S10. CgNluc performance in fecal material. Data from Figure 5c plotted as the fold-change in luminescence for varying concentrations of PPEP-1 (in PBS or fecal material) compared to signal in the absence of PPEP-1. All samples were diluted to 10 nM for imaging.

References

1. Quan, J.; Tian, J. Circular polymerase extension cloning for high-throughput cloning of complex and combinatorial DNA libraries. *Nat. Protoc. 2011 62* **2011**, *6*, 242–251, doi:10.1038/nprot.2010.181.

2. Gibson, D.G.; Young, L.; Chuang, R.-Y.; Venter, J.C.; Hutchison, C.A.; Smith, H.O. Enzymatic assembly of DNA molecules up to several hundred kilobases. *Nat. Methods 2009 65* **2009**, *6*, 343–345, doi:10.1038/nmeth.1318.

3. Trott, O.; Olson, A.J. AutoDock Vina: Improving the speed and accuracy of docking with a new scoring function, efficient optimization, and multithreading. *J. Comput. Chem.* **2009**, *31*, NA-NA, doi:10.1002/jcc.21334.

4. Morris, G.M.; Ruth, H.; Lindstrom, W.; Sanner, M.F.; Belew, R.K.; Goodsell, D.S.; Olson, A.J. Software news and updates AutoDock4 and AutoDockTools4: Automated docking with selective receptor flexibility. *J. Comput. Chem.* **2009**, *30*, 2785–2791, doi:10.1002/jcc.21256.

Table S1. Primers used in plasmid construction.

Plasmid	Vector	Insert	Primers (5' - 3')
CgNBiT1	pCold	LgBiT	CATCATATCGAAGGTAGGCATATGGTCTTCACACTCGAAGATTTCGTTGG
			GAGGGTTGACGACCGAACCTCCCCCGCTGTTGATGGTTACTCGGAACAG
		SmBiT	GTCGTCAACCCTCCTGTACCACCGTCCGGGGGCGGTAATGTATCGGGCTGGCG
			GGTCGACAAGCTTGAATTCTTAGTTACTAATTTTCTTAAAC
CgNBiT2	pCold	LgBiT	AGTAACGTCAACCCCCCTGTTCCCCCAGTCTTCACACTCGAAGATTTCGTT
			AGGTCGACAAGCTTGAATTCTTAGCTGTTGATGGTTACTCGGAACAG
		SmBiT	CATATCGAAGGTAGGCATATGAATGTATCGGGCTGGCGCTTGTTTAAGAAAATTAGTAAC
			GAAGACTGGGGGAACAGGGGGGTTGACGTTACTAATTTTCTTAAACAAGCGCCAGCCC
CgNBiT3	pCold	LgBiT	GAGTCCGTCAACCCCCCTGTTCCCCCAGTCTTCACACTCGAAGATTTCGTT
			AGGTCGACAAGCTTGAATTCTTAGCTGTTGATGGTTACTCGGAACAG
		SmBiT	CATATCGAAGGTAGGCATATGGTTACGGGATACCGCTTATTCGAGAAAATTTGTGAAGACTGG
			TGTGAAGACTGGGGGAACAGGGGGGTTGACCAAAATTTTCTCGAATAAGCGGTATCCCGT
Invasin - NBiT	pCold	LgBiT	CCCTTTGCGCGTTATCTATTGTCTTCACACTCGAAGATTTCGTT
			GGTCGACAAGCTTGAATTCTTA GCTGTTGATGGTTACTCGGAACAG
		Invasin	GAGGTGAATCCCCCAGTACCCGATAGTGTGACTGTCCAACAAC
			AACGAAATCTTCGAGTGTGAAGACAATAGATAACGCGCAAAGGG
		SmBiT	CATCATATCGAAGGTAGGCATGTCGAAGGGTATCGTTTGTTTGAGAAAATCAGCGAGG
			GTTGTTGGACAGTCACACTATCGGGTACTGGGGGATTCACCTCGCTGATTTTCTCAAACA
CgSmBiT	pET	K5 Coil - SmBiT99	GTTTAACTTTAAGAAGGAGATATACCATGGGCGGCAGCAAGGTGTC
			CGGGTTTACCGAAATTTTCTCAAAAAGGCGATAGCCTGTCACTTCTTTCAGCGCGCTCA
		SmBiT99 - E5 Coil	GAGAAAATTTCGGTAAACCCGCCGGTGCCGCCCGAAGTGAGCGCGCTGGAAAAAG
			TTAGCAGCCGGATCTCATTAGTGATGGTGATGGTGATGTTTTTCAAGCGCAGAGACTTCC
CgLgBiT	pET	E5 Coil	GTTTAACTTTAAGAAGGAGATATACCATGGAAGTGAGCGCGCTGGAAAAAG
			AACGAAATCTTCGAGTGTGAAGACTTTTTCAAGCGCAGAGACTTCCTTCT
		LgBIT	AGAAGGAAGTCTCTGCGCTTGAAAAAGTCTTCACACTCGAAGATTTCGTT
			GGGCGGCACCGGCGGGTTTACGCTGTTGATGGTTACTCGGAACAG
		K5 Coil	AGCGTAAACCCGCCGGTGCCGCCCATGGGCGGCAGCAAGGTGTC
			TTAGCAGCCGGATCTCATTAGTGATGGTGATGGTGATGTTCTTTCAGCGCGCTCACT
PPEP-1	pET	PPEP-1	CATCACCATCACGAAAATCTTTATTTTCAAGGCGACAGCACCACTATTCAACAG’
			CAGTGGTGGTGGTGGTGGTGATTATTATTTCGCCAAGTTTTG
CgNluc	pET	CgNluc	TTTGTTTAACTTTAAGAAGGAGATATACTCGAGATGGTCTTCACACTCGAAGATTTCG
			GCGAACGCATTCTGGCGGTAAACCCGCCGGTGCCGCCCGGTGGTGGTGGTTCTGGTGGTG
		CgNluc Ext.	GTGGTGGTTCTGGTGGTGGAGGAAGCCATCACCATCACCATCACTAAATAGAATTCTAAT
Nluc – RH – IRES – eGFP	pCDNA	Nluc - RH	ACGACTCACTATAGGGAGACCCAAGCTTCGCCACCATGGTCTTCACACTCGAAGATTTCG
			GCGAACGCATTCTGGCGCGTCATTAAACGTTACTGGCCGAAGCCG
		IRES - eGFP	ACGTTACTGGCCGAAGCC
			TCGGCATGGACGAGCTGTACAAGTAGGAATTCTGCAGATATCCATCACACTGGCGGCCGC
Nluc – G_4S – IRES – eGFP	pCDNA	Nluc– G_4S	ACGACTCACTATAGGGAGACCCAAGCTTCGCCACCATGGTCTTCACACTCGAAGATTTCG
			GCGAACGCATTCTGGCGGGTGGTGGTGGTTCTTAAACGTTACTGGCCGAAGCCG
		IRES - eGFP	ACGTTACTGGCCGAAGCC
			TCGGCATGGACGAGCTGTACAAGTAGGAATTCTGCAGATATCCATCACACTGGCGGCCGC
Nluc – G_4S_{x2} – IRES – eGFP	pCDNA	Nluc – G_4S_{x2}	ACGACTCACTATAGGGAGACCCAAGCTTCGCCACCATGGTCTTCACACTCGAAGATTTCG
			CTGTGCGAACGCATTCTGGCGGGTGGTGGTGGTTCTGGTGGTGGTGGTTCT
		IRES - eGFP	GGTGGTGGTGGTTCTGGTGGTGGTGGTTCTTAGACGTTACTGGCCGAAGCCGC
			TCGGCATGGACGAGCTGTACAAGTAGGAATTCTGCAGATATCCATCACACTGGCGGCCGC
Nluc – G_4S_{x5} – IRES – eGFP	pCDNA	Nluc – G_4S_{x5}	ACGACTCACTATAGGGAGACCCAAGCTTCGCCACCATGGTCTTCACACTCGAAGATTTCG
			GAACGCATTCTGGCGGGCGGAGGCGGGTCTGGGGGCGGAGGCTCTGGCGGCGGTGGTTCT
		Nluc – G_4S_{x5} Ext.	GGCGGCGGTGGTTCTGGAGGAGGTGGTTCTGGCGGTGGGGGTTCTTAAACGTTACTGGCC
		IRES - eGFP	GGCGGTGGGGGTTCTTAAACGTTACTGGCCGAAGCC
			TCGGCATGGACGAGCTGTACAAGTAGGAATTCTGCAGATATCCATCACACTGGCGGCCGC
Nluc – EAAAK – IRES – eGFP	pCDNA	Nluc-EAAAK	ACGACTCACTATAGGGAGACCCAAGCTTCGCCACCATGGTCTTCACACTCGAAGATTTCG
			TGTGCGAACGCATTCTGGCGGAAGCTGCCGCAAAGTAAACGTTACTGGCCGAAGCC
		IRES - eGFP	ACGTTACTGGCCGAAGCC
			TCGGCATGGACGAGCTGTACAAGTAGGAATTCTGCAGATATCCATCACACTGGCGGCCGC
Nluc – EAAAK – G_4S_{x2} – IRES – eGFP	pCDNA	Nluc-EAAAK – G_4S_{x2}	ACGACTCACTATAGGGAGACCCAAGCTTCGCCACCATGGTCTTCACACTCGAAGATTTCG
			GAACGCATTCTGGCGGAAGCTGCCGCAAAGGGCGGAGGCGGGTCTGGCGGTGGGGGTTCT
		IRES - eGFP	GGCGGTGGGGGTTCTTAAACGTTACTGGCCGAAGCC
			TCGGCATGGACGAGCTGTACAAGTAGGAATTCTGCAGATATCCATCACACTGGCGGCCGC
